# Reading Times of Common Musculoskeletal MRI Examinations: A Survey Study

**DOI:** 10.3390/tomography10090112

**Published:** 2024-09-20

**Authors:** Robert M. Kwee, Asaad A. H. Amasha, Thomas C. Kwee

**Affiliations:** 1 Zuyderland Medical Center, 6419 PC Heerlen, The Netherlands; 2 University Medical Center Groningen, 9713 GZ Groningen, The Netherlands; a.a.amasha@student.rug.nl (A.A.H.A.); t.c.kwee@umcg.nl (T.C.K.)

**Keywords:** workload, radiology, magnetic resonance imaging

## Abstract

Background: The workload of musculoskeletal radiologists has come under pressure. Our objective was to estimate the reading times of common musculoskeletal MRI examinations. Methods: A total of 144 radiologists were asked to estimate reading times (including interpretation and reporting) for MRI of the shoulder, elbow, wrist, hip, knee, and ankle. Multivariate linear regression analyses were performed. Results: Reported median reading times with interquartile range (IQR) for the shoulder, elbow, wrist, hip, knee, and ankle were 10 (IQR 6–14), 10 (IQR 6–14), 11 (IQR 7.5–14.5), 10 (IQR 6.6–13.4), 8 (IQR 4.6–11.4), and 10 (IQR 6.5–13.5) min, respectively. Radiologists aged 35–44 years reported shorter reading times for the shoulder (β coefficient [β] = B-3.412, *p* = 0.041), hip (β = −3.596, *p* = 0.023), and knee (β = −3.541, *p* = 0.013) than radiologists aged 45–54 years. Radiologists not working in an academic/teaching hospital reported shorter reading times for the hip (β = −3.611, *p* = 0.025) and knee (β = −3.038, *p* = 0.035). Female radiologists indicated longer reading times for all joints (β of 2.592 to 5.186, *p* ≤ 0.034). Radiologists without musculoskeletal fellowship training indicated longer reading times for the shoulder (β = 4.604, *p* = 0.005), elbow (β = 3.989, *p* = 0.038), wrist (β = 4.543, *p* = 0.014), and hip (β = 2.380, *p* = 0.119). Radiologists with <5 years of post-residency experience indicated longer reading times for all joints (β of 5.355 to 6.984, *p* ≤ 0.045), and radiologists with 5–10 years of post-residency experience reported longer reading time for the knee (β = 3.660, *p* = 0.045) than those with >10 years of post-residency experience. Conclusions: There is substantial variation among radiologists in reported reading times for common musculoskeletal MRI examinations. Several radiologist-related determinants appear to be associated with reading speed, including age, gender, hospital type, training, and experience.

## 1. Introduction

MRI has emerged as an indispensable tool in musculoskeletal imaging [1]. The acquisition speed, the number of images that comprise a typical MRI examination, and the overall number of musculoskeletal MRI examinations have increased considerably over the past decades [1,2,3]. Reading each image of an MRI examination in less than a second has become the norm [4]. Meanwhile, there is a global shortage of radiologists [5]. These developments put an increasing workload burden on radiologists. A survey conducted in 2016 revealed that the prevalence and intensity of burnout among musculoskeletal radiologists are higher compared to radiologists in general and compared to other physicians [6]. Physician productivity and workload are typically measured using Relative Value Units (RVUs). However, it has been shown that the current RVU system is inaccurate and underestimates the work effort of the radiologist in terms of time, especially in musculoskeletal imaging [7]. This inaccuracy contributes to an imbalanced workload that may contribute to burnout [7] and heightens the risk of diagnostic errors [7,8,9,10,11]. A more accurate representation of a radiologist’s workload could be achieved through a time-based metric rather than relying solely on RVUs [7]. Therefore, the primary objective of our study was to estimate reading times of common musculoskeletal MRI examinations, aiming to provide a more precise measure of the workload and contribute to a better understanding of radiologists’ efforts in this specialized field.

## 2. Methods

### 2.1. Study Design and Participants

The survey study received approval from the Medical Ethics Review Committee (name blinded for review). Musculoskeletal imaging experts who had published in Skeletal Radiology, Seminars in Musculoskeletal Radiology, or Radiology between 2009 and 2023 and with an available corresponding email address were invited to participate. The survey focused on gathering insights into the time it takes to read common musculoskeletal MRI examinations. The initial email request to the corresponding authors was dispatched at the beginning of February 2024. Six reminder emails were sent at one-week intervals. Corresponding authors within the circle of acquaintances of the investigators were excluded from participation.

### 2.2. Survey

Participants were first asked if they were radiologists. The survey then continued with questions related to participants’ demographics, hospital type, training, and professional experience. Participants were also queried whether they utilized an artificial intelligence (AI)-based tool to help with the interpretation of MRI scans. Subsequently, participants were asked to estimate the approximate time it takes them to independently interpret and report MRI examinations of the shoulder, elbow, wrist, hip, knee, and ankle (hereinafter referred to as reading time) in minutes. The survey, available in Supplementary File S1, was accessible through a Qualtrics software-generated weblink (Available online: https://www.qualtrics.com/ (accessed on 4 February 2024), Qualtrics, Provo, UT, USA). The questionnaire was completed anonymously and is not traceable to individual participants.

### 2.3. Data Analysis

Participants who were not radiologists were excluded from the analysis. Box-and-whisker plots were constructed to display the distribution of reported reading times for each body part. Multivariate linear regression analyses were performed to determine the association of reading time with the following variables: age, gender, type of hospital (academic/teaching vs. non-academic/non-teaching), training (fellowship-trained vs. non-fellowship-trained in musculoskeletal radiology), and years of post-residency experience. Categories with 5 or fewer counts were not entered in the linear regression analyses. Categories with the largest number of counts were used as references. *p*-values less than 0.05 were considered statistically significant. Statistical analyses were executed using IBM Statistical Package for the Social Sciences (SPSS) version 28.

## 3. Results

### 3.1. Respondents

Of the 2264 corresponding authors with a valid email address, 201 responded to the survey (8.9% response rate). There were 41 respondents who were excluded because they were not radiologists. Another 16 respondents were excluded because they did not report any reading time. Eventually, 144 radiologists were included in our study. Their characteristics are displayed in Table 1. Most radiologists were aged 35 to 64 years (87.5%), male (75.0%), and worked in Europe (45.8%) or North America (33.3%). Most radiologists worked in an academic/teaching hospital (81.9%), were musculoskeletal fellowship-trained (82.6%), and had more than 10 years of post-residency experience in interpreting and reporting musculoskeletal MRI examinations (74.3%). Only a minority of the radiologists were using an AI-based tool to help with the interpretation of musculoskeletal MRI examinations (6.3%).

### 3.2. Reading Time of Common Musculoskeletal MRI Examinations

**Figure 1:**
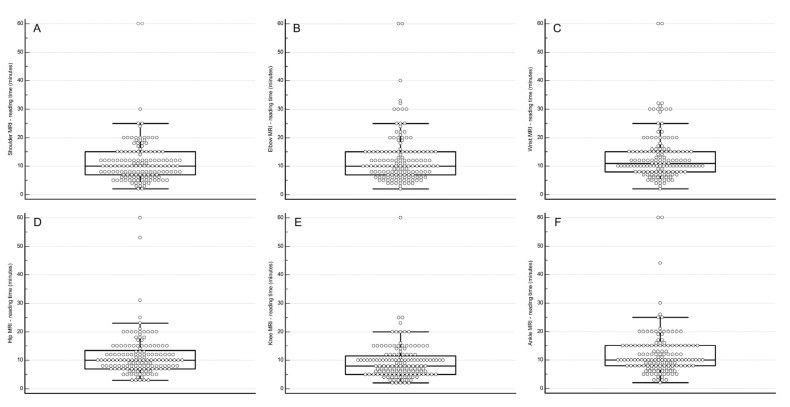
Box-and-whisker plot showing the distribution of reported reading times for MRI of the shoulder (**A**), elbow (**B**), wrist (**C**), hip (**D**), knee (**E**), and ankle (**F**). Two radiologists account for the extreme outliers, i.e., reported reading times of 53 to 60 min.

There was substantial inter-individual variation in reading times for each joint, as shown by the box-and-whisker plots (Figure 1).

- Shoulder: median 10 min (interquartile range [IQR] 6–14, range 2–60).
- Elbow: median 10 min (IQR 6–14, range 2–60).
- Wrist: median 11 min (IQR 7.5–14.5, range 2–60).
- Hip: median 10 min (IQR 6.6–13.4, range 3–60).
- Knee: median 8 min (IQR 4.6–11.4, range 2–60).
- Ankle: median 10 min (IQR 6.5–13.5, range 2–60).

### 3.3. Determinants of Reading Time

The associations between several characteristics of the radiologists and reported reading time of common musculoskeletal MRI examinations are displayed in Table 2.

Age 35–44 years (reference category: 45–54 years) was significantly associated with a shorter reading time of the shoulder (β coefficient [β] of −3.412, 95% confidence interval [CI]: 6.676 to −0.147, *p* = 0.041), hip (β of −3.596, 95% CI: 6.693 to −0.499, *p* = 0.023), and knee (β of −3.541, 95% CI: −6.312 to −0.770, *p* = 0.013).

Female gender was significantly associated with a longer reading time of the shoulder (β of 5.186, 95% CI: 2.365 to 8.007, *p* < 0.001), elbow (β of 4.229, 95% CI: 0.863 to 7.595, *p* = 0.014), wrist (β of 3.980, 95% CI: 0.749 to 7.210, *p* = 0.016), hip (B 3.704, 95% CI: 1.028 to 6.381, *p* = 0.007), knee (β of 2.592, 95% CI: 0.198 to 4.986, *p* = 0.034), and ankle (β of 3.329, 95% CI: 0.220 to 6.438, *p* = 0.036).

Not working in an academic/teaching hospital was significantly associated with a shorter reading time of the hip (β of −3.611, 95% CI: −6.759 to −0.464, *p* = 0.025) and knee (β of −3.038, 95% CI: −5.854 to −0.222, *p* = 0.035).

Not having completed a musculoskeletal radiology fellowship was significantly associated with a longer reading time of the shoulder (β of 4.604, 95% CI: 1.441 to 7.766, *p* = 0.005), elbow (β of 3.989, 95% CI: 0.215 to 7.763, *p* = 0.038), wrist (β of 4.543, 95% CI: 0.921 to 8.165, *p* = 0.014), and hip (β of 2.380, 95% CI: −0.621 to 5.380, *p* = 0.119).

Post-residency experience of <5 years (reference category: post-residency experience of >10 years) was significantly associated with a longer reading time of the shoulder (β of 5.837, 95% CI: 1.216 to 10.458, *p* = 0.014), elbow (β of 5.639, 95% CI:0.125 to 11.153, *p* = 0.045), wrist (β of 7.214, 95% CI: 1.922 to 12.506, *p* = 0.008), hip (β of 6.948, 95% CI: 2.564 to 11.332, *p* = 0.002), knee (β of 5.355, 95% CI: 1.433 to 9.277, *p* = 0.008), and ankle (β of 6.162, 95% CI: 1.069 to 11.254, *p* = 0.018). Post-residency experience of 5–10 years was significantly associated with a longer reading time of the knee (β of 3.660, 95% CI: 0.082 to 7.238, *p* = 0.045).

## 4. Discussion

The workload of musculoskeletal radiologists has come under pressure. Our study provided reading times for common musculoskeletal MRI examinations (shoulder, elbow, wrist, hip, knee, and ankle) based on the estimates of 144 radiologists. Median reported reading times ranged between 8 min (knee) and 11 min (wrist). There was substantial inter-individual variation in reading times for each joint. Our study data can be utilized to assess workload and may also serve as a benchmark for reading speed for residents and fellows aspiring to specialize in musculoskeletal radiology.

To our knowledge, there are only a few studies that investigated reading times for common cross-sectional radiological examinations. This topic has recently come to attention in the field of neuroradiology and was also driven by the issue of increased workload [12]. This 2022 survey indicated that a median of 32 CT and/or MRI examinations (IQR, 23–36) could be reasonably and safely independently read in a regular full clinical day [12]. There is a lack of studies in the field of musculoskeletal radiology on this topic. In 2015, a working group by the Royal Australian and New Zealand College of Radiologists (RANZCR) estimated time-based metrics for reporting medical imaging exams [13]. The estimated reporting times of common musculoskeletal MRI exams varied between 16 min (shoulder, elbow, hip, and knee) and 18 min (wrist and ankle) [13], which was longer than the median reported reading times in our study (8 to 11 min). This difference can be explained because the RANZCR working group also included ascribable tasks other than interpreting and reporting (such as interpretation and clarification of requests, examination protocolling, supervision of technical staff, and phone calls to referrers), which were not included in the present study [13]. Moreover, the RANZCR working group estimates were only aimed at an academic/teaching hospital setting [13]. The RANZCR working group did not analyze determinants that may influence reading time. Our study showed that there is a substantial variation in reading times that is associated with different characteristics of radiologists. Radiologists aged 35–44 years reported shorter reading times for the shoulder (β of −3.412, *p* = 0.041), hip (β of −3.596, *p* = 0.023), and knee (β of −3.541, *p* = 0.013) compared to radiologists aged 45–54 years. We could not explain the cause of this difference. Strikingly, female radiologists reported a significantly longer reading time for all common musculoskeletal MRI examinations (β of 2.592 to 5.186, *p* ≤ 0.034). The cause is also unclear and needs further investigation. Radiologists not working in an academic/teaching hospital reported significantly shorter reading times for the hip (β of −3.611, *p* = 0.025) and knee (β of −3.038, *p* = 0.035). It could be possible that they have more routine in reading these studies due to higher volumes, or it could be possible that the hip and knee MRI examinations they read are generally less complicated. However, these assumptions remain speculative. Radiologists who did not pursue a musculoskeletal radiology fellowship reported longer reading times for the shoulder (β of 4.604, *p* = 0.005), elbow (β of 3.989, *p* = 0.038), wrist (β of 4.543, *p* = 0.014), and hip (β of 2.380, *p* = 0.119). The least experienced radiologists (<5 years of post-residency experience) also reported longer reading times for all joints (β of 5.355 to 6.984, *p* ≤ 0.045), which makes complete sense.

Artificial intelligence (AI) has the potential to significantly enhance the interpretation of musculoskeletal imaging studies, which could lead to a reduction in the workload faced by radiologists and other healthcare professionals involved in diagnostic imaging [14,15]. By assisting in the analysis of complex imaging data, AI could streamline the diagnostic process, allowing healthcare providers to handle a higher volume of cases with greater efficiency. However, before AI can be widely adopted into widespread clinical practice, it must undergo extensive testing and validation to ensure its accuracy, reliability, and safety in various clinical settings [14,15]. This need for further validation explains why, as reported in our study, only 6.3% of respondents currently utilize AI tools to aid in the interpretation of musculoskeletal MRI examinations. These low adoption rates reflect the cautious approach the medical community is taking to ensure that AI applications meet the rigorous standards required for clinical use. The findings from our study, which include detailed reporting times for common musculoskeletal MRI examinations, could serve as a valuable benchmark for future research focused on optimizing AI-driven workflows. Studies aiming to increase the speed of interpretation and reporting through AI can use these data as a reference point to measure their effectiveness. Furthermore, AI has the potential to increase reporting speed by improving hanging protocols, report generation, and communication [16,17]. Beyond image interpretation and processing, AI could offer significant benefits to the radiology workflow, easing the burden on radiologists (and MRI technicians) by enhancing non-interpretive tasks. These tasks include patient scheduling, designing optimal imaging protocols, reducing MRI acquisition and reconstruction times, and improving image quality [16,17].

Our study has some limitations. First, reading time was not measured but based on estimates by radiologists. Further research is necessary to prospectively record “actual” reading times. Second, our analysis was based on interpreting and reporting time only, whereas there may be more ascribable times to each MRI examination [13]. In addition, non-interpretative tasks and task-switching events can consume up to 50% of a radiologist’s working time [18]. Third, we did not determine the proportion of radiologists who focus primarily on MSK imaging in their current practice, and we also did not determine the proportion of MSK cases in their overall workload. Both determinants may influence reading time, but it remains unclear to what extent. Fourth, although fatigue occurs among radiologists and may affect diagnostic accuracy [19], we did not assess how many MRI examinations could be reasonably and safely performed per working day. However, maximum reading volumes per day largely depend on the type of practice the radiologist is working in, are likely highly individual-dependent, and may be less generalizable than reading time per MRI examination. Fifth, we did not perform subgroup analyses to determine the effect of the use of AI on reading times because our survey did not record for which MRI examinations (e.g., shoulder, elbow, wrist, hip, knee, or ankle) AI was used by the respondents. In addition, we did not perform any subgroup analyses according to continent because individual countries in a single continent may be very different, and we did not have any a priori hypothesis either as to why reporting times should be faster or slower in a certain region.

## 5. Conclusions

In conclusion, there is substantial variation among radiologists in reported reading times for common musculoskeletal MRI examinations. Several radiologist-related determinants appear to be associated with reading speed, including age, gender, hospital type, training, and experience.

## Figures and Tables

**Table 1:** Characteristics of the 144 radiologists who participated in the survey.

	Category	Number and %
Age distribution	25–34 years	n = 5 (3.5%)
	35–44 years	n = 39 (27.1%)
	45–54 years	n = 48 (33.3%)
	55–64 years	n = 39 (27.1%)
	>65 years	n = 13 (9.0%)
Gender	Male	n = 108 (75.0%)
	Female	n = 35 (24.3%)
	Other	n = 1 (0.7%)
Continent	Europe	n = 66 (45.8%)
	North America	n = 48 (33.3%)
	Asia	n = 19 (13.2%)
	South America	n = 5 (3.5%)
	Australia	n = 5 (3.5%)
	Africa	n = 1 (0.7%)
Working in an academic/teaching hospital	Yes	n = 118 (81.9%)
	No	n = 26 (18.1%)
Fellowship-trained musculoskeletal radiologist	Yes	n = 119 (82.6%)
	No	n = 25 (17.4%)
Post-residency experience in interpreting and reporting musculoskeletal MRI examinations	<5 years	n = 13 (9.0%)
	5–10 years	n = 24 (16.7%)
	>10 years	n = 107 (74.3%)
Currently using an AI-based tool to help with interpretation of musculoskeletal MRI examinations	Yes	n = 9 (6.3%)
	No	n = 135 (93.8%)

**Table 2:** Association of variables with an independent reading time of common musculoskeletal MRI examinations (β coefficients, significant *p*-values are displayed in bold).

Variable	Category	Shoulder	Elbow	Wrist	Hip	Knee	Ankle
Age ^1^	35–44 years	**−3.412 (−6.676 to −0.147)** ***p* = 0.041**	−2.120 (−6.016 to 1.775) *p* = 0.284	−1.857 (−5.595 to 1.882) *p* = 0.328	**−3.596 (−6.693 to −0.499) *p* = 0.023**	**−3.541 (−6.312 to −0.770 *p* = 0.013**	−3.276 (−6.874 to 0.322) *p* = 0.074
	55–64 years	−0.850 (−3.919 to 2.219) *p* = 0.585	−0.859 (−4.522 to 2.803) *p* = 0.643	0.041 (−3.474 to 3.556) *p* = 0.981	−1.495 (−4.407 to 1.416) *p* = 0.312	−1.370 (−3.975 to 1.225) *p* = 0.300	−0.917 (−4.299 to 2.466) *p* = 0.593
	>65 years	0.644 (−3.855 to 5.143) *p* = 0.778	−1.010 (−6.378 to 4.358) *p* = 0.710	−0.405 (−5.557 to 4.747) *p* = 0.877	0.144 (−4.124 to 4.412) *p* = 0.947	−0.188 (−4.006 to 3.630) *p* = 0.923	0.223 (−4.735 to 5.181) *p* = 0.929
Gender ^2^	Female	**5.186 (2.365 to 8.007) *p* < 0.001**	**4.229 (0.863 to 7.595) *p* = 0.014**	**3.980 (0.749 to 7.210) *p* = 0.016**	**3.704 (1.028 to 6.381) *p* = 0.007**	**2.592 (0.198 to 4.986) *p* = 0.034**	**3.329 (0.220 to 6.438) *p* = 0.036**
Working in an academic/teaching hospital ^3^	No	−3.232 (−6.550 to 0.085) *p* = 0.056	−4.086 (−8.045 to −0.127) *p* = 0.043	−3.722 (−7.522 to 0.077) *p* = 0.055	**−3.611 (−6.759 to −0.464) *p* = 0.025**	**−3.038 (−5.854 to −0.222) *p* = 0.035**	−2.753 (−6.410 to 0.904) *p* = 0.139
Fellowship-trained musculoskeletal radiologist ^4^	No	**4.604 (1.441 to 7.766) *p* = 0.005**	**3.989 (0.215 to 7.763) *p* = 0.038**	**4.543 (0.921 to 8.165) *p* = 0.014**	**2.380 (−0.621 to 5.380) *p* = 0.119**	1.447 (−1.238 to 4.131) *p* = 0.288	2.821 (−0.665 to 6.306) *p* = 0.112
Post-residency experience in interpreting and reporting musculoskeletal MRI examinations ^5^	<5 years	**5.837 (1.216 to 10.458) *p* = 0.014**	**5.639 (0.125 to 11.153) *p* = 0.045**	**7.214 (1.922 to 12.506) *p* = 0.008**	**6.948 (2.564 to 11.332) *p* = 0.002**	**5.355 (1.433 to 9.277) *p* = 0.008**	**6.162 (1.069 to 11.254) *p* = 0.018**
	5–10 years	3.022 (−0.750 to 6.794) *p* = 0.115	2.172 (−2.329 to 6.673) *p* = 0.342	2.578 (−1.742 to 6.897) *p* = 0.240	**3.660 (0.082 to 7.238)** ***p* = 0.045**	2.159 (−1.042 to 5.360) *p* = 0.184	3.788 (−0.369 to 7.945) *p* = 0.074

Notes: ^1^ 45–54 years was used as a reference category; ^2^ male gender was used as a reference category; ^3^ academic/teaching hospital was used as a reference category; ^4^ fellowship-trained musculoskeletal radiologist was used as a reference category; ^5^ post-residency experience > 10 years was used as a reference category.

## Data Availability

Data are available upon reasonable request, by contacting the corresponding author.

## Supplementary Materials

The following supporting information can be downloaded at: https://www.mdpi.com/article/10.3390/tomography10090112/s1, File S1: Survey questionnaire.
