# Supplementary material for: Reading Times of Common Musculoskeletal MRI Examinations: A Survey Study
Source: Tomography. 2024 Sep 20;10(9):1527–33. doi: 10.3390/tomography10090112 (PMC11435788; doi:10.3390/tomography10090112)
Supplement: Supplementary file 1 [file tomography-10-00112-s001.zip › tomography-3178670-supplementary.pdf]

Q1 Are you a radiologist?

- Yes (1)
- No (2)

Display This Question:

If Are you a radiologist? = Yes

Q2 How old are you?

- <25 years (2)
- 25-34 years (3)
- 35-44 years (4)
- 45-54 years (5)
- 55-64 years (6)
- 65+ years (7)

Display This Question:

If Are you a radiologist? = Yes

Q3 What is your gender?

- Male (1)
- Female (2)
- Other (3)

Display This Question:

If Are you a radiologist? = Yes

Q4 In which country do you work?

- ○ Argentina (1)
- ○ Australia (2)
- ○ Austria (3)
- ○ Belgium (4)
- ○ Brazil (5)
- ○ Canada (6)
- ○ China (7)
- ○ Denmark (8)
- ○ Egypt (9)
- ○ Finland (10)
- ○ France (11)
- ○ Germany (12)
- ○ Greece (13)
- ○ India (31)
- ○ Ireland (14)
- ○ Italy (15)
- ○ Japan (16)
- ○ Korea (17)
- ○ New Zealand (18)
- ○ Norway (19)
- ○ Poland (20)

- Portugal (21)
- Saudi Arabia (22)
- Spain (23)
- Sweden (24)
- Switzerland (25)
- The Netherlands (26)
- Turkey (27)
- United Kingdom (28)
- United States of America (29)
- Other, namely: (30) ____

Display This Question:

If Are you a radiologist? = Yes

Q5 Are you working in an academic/teaching hospital?

- Yes (1)
- No (2)

Display This Question:

If Are you a radiologist? = Yes

Q6 Are you a fellowship-trained musculoskeletal radiologist?

- Yes (1)
- No (2)

Display This Question:

If Are you a radiologist? = Yes

Q7 How many years of post-residency experience do you have in interpreting and reporting musculoskeletal MRI scans?

- <5 years (1)
- 5 to 10 years (2)
- >10 years (3)

Page Break

Display This Question:

If Are you a radiologist? = Yes

Q8 Do you currently use an artificial intelligence (AI)-based tool to help with interpretation of musculoskeletal MRI scans?

- Yes (1)
- No (2)

Page Break

Display This Question:

If Are you a radiologist? = Yes

Q9 On the basis of your expertise and experience, please estimate below how many minutes it approximately takes to independently interpret and report each of the following MRI scans:

	Time in minutes<br>0 4 8 12 16 20 24 28 32 36 40 44 48 52 56 60
MRI of the shoulder ()	
MRI of the elbow ()	
MRI of the wrist ()	
MRI of the hip ()	
MRI of the knee ()	
MRI of the ankle ()	

Display This Question:

If Are you a radiologist? = Yes

Q10 Please feel free to add any narrative comments:

____

____

____

____

____
